# Functional avoidance‐based intensity modulated proton therapy with 4DCT derived ventilation imaging for lung cancer

**DOI:** 10.1002/acm2.13323

**Published:** 2021-06-22

**Authors:** Jingjing M. Dougherty, Edward Castillo, Richard Castillo, Austin M. Faught, Mark Pepin, Sean S. Park, Chris J. Beltran, Thomas Guerrero, Inga Grills, Yevgeniy Vinogradskiy

**Affiliations:** ^1^ Department of Radiation Oncology Mayo Clinic Jacksonville FL USA; ^2^ Department of Computational and Applied Mathematics Rice University Houston TX USA; ^3^ Department of Radiation Oncology Beaumont Health System Royal Oak MI USA; ^4^ Department of Radiation Oncology Emory University Atlanta GA USA; ^5^ Department of Radiation Oncology St. Jude Children's Research Hospital Memphis TN USA; ^6^ Department of Radiation Oncology Mayo Clinic Rochester MN USA; ^7^ Department of Radiation Oncology University of Colorado School of Medicine Aurora CO USA

**Keywords:** 4DCT, functional lung imaging, IMPT, lung toxicity, lung ventilation, proton motion interplay, proton therapy, radiation pneumonitis

## Abstract

The primary objective is to evaluate the potential dosimetric gains of performing functional avoidance‐based proton treatment planning using 4DCT derived ventilation imaging. 4DCT data of 31 patients from a prospective functional avoidance clinical trial were evaluated with intensity modulated proton therapy (IMPT) plans and compared with clinical volumetric modulated arc therapy (VMAT) plans. Dosimetric parameters were compared between standard and functional plans with IMPT and VMAT with one‐way analysis of variance and post hoc paired student t‐test. Normal Tissue Complication Probability (NTCP) models were employed to estimate the risk of two toxicity endpoints for healthy lung tissues. Dose degradation due to proton motion interplay effect was evaluated. Functional IMPT plans led to significant dose reduction to functional lung structures when compared with functional VMAT without significant dose increase to Organ at Risk (OAR) structures. When interplay effect is considered, no significant dose degradation was observed for the OARs or the clinical target volume (CTV) volumes for functional IMPT. Using fV20 as the dose metric and Grade 2+ pneumonitis as toxicity endpoint, there is a mean 5.7% reduction in Grade 2+ RP with the functional IMPT and as high as 26% in reduction for individual patient when compared to the standard IMPT planning. Functional IMPT was able to spare healthy lung tissue to avoid excess dose to normal structures while maintaining satisfying target coverage. NTCP calculation also shows that the risk of pulmonary complications can be further reduced with functional based IMPT.

## 1 INTRODUCTION

### 1.1 Functional avoidance radiation therapy for definitive lung cancer

The regional function heterogeneity of lung tissues has long been exploited in the design of external beam radiation therapy treatment planning using single photon emission tomography (SPECT) with radiopharmaceutical aerosols.^1^, ^2^, ^3^ Positron emission tomography (PET) paired with a positron emitting inert gas is another reported ventilation imaging method.^4^, ^5^ Hyperpolarized xenon or helium magnetic resonance imaging (MRI) techniques have also been reported to generate pulmonary ventilation information.^6^, ^7^, ^8^ With the advent of 4DCT‐based pulmonary ventilation calculation, the changes in the local air content are converted into pulmonary functional images to guide the placing and optimization of radiation treatment beams.^9^, ^10^, ^11^, ^12^, ^13^, ^14^ Currently, there are multiple National Institutes of Health (NIH) funded clinical trials (NCT02528942, NCT02308709, and NCT02843568) that aim to investigate the efficacy of the functional avoidance‐based treatment planning techniques.

### 1.2 Proton beam therapy for lung cancer

The ability of proton beams to stop in tissue within a finite range provides its dosimetric superiority over conventional external beam photon therapy. However, complexity in proton treatment planning increases when considering uncertainties in proton range, patient daily setup, physiological changes of the tumor, CT density conversion, and tumor motion. In a recent large‐cohort retrospective comparison study, the National Cancer Database was queried to analyze the outcomes and predictors associated with proton therapy for stage I‐IV non‐small cell lung cancer (NSCLC) patients; better 5‐year overall survival was reported for proton radiation therapy^15^; better overall survival rates and tolerable toxicities were also reported for stage II‐III NSCLC when using proton therapy with concurrent chemotherapy.^16^, ^17^, ^18^ More clinical trials are available to investigate the effect of proton beam therapy on local control rates, toxicity, and survival improvement for patients with early or advanced stage lung cancer (NCT00875901, NCT01629498, NCT01993810, NCT03132532). With more proton centers employing scanning pencil beam methods for treatment delivery and plan optimization, the interplay effect of tumor motion and proton spot arrangement has gone through vigorous numerical simulations,^19^, ^20^, ^21^, ^22^, ^23^ measurement validations,^24^, ^25^ and methods to manage it have been clinically implemented.^26^, ^27^, ^28^, ^29^ The layer or volumetric dose repainting techniques are the most investigated and applied methodologies in mitigating dose degradation caused by the motion interplay effect due to relatively easy clinical implementation within commercial treatment planning systems.

The purpose of this study is to investigate the potential dosimetric gains and toxicity reduction for the 4DCT‐based functional avoidance treatment planning technique using robustly optimized intensity modulated proton therapy (IMPT). Clinical data from a phase II photon functional avoidance study were exported to perform an in‐silico comparison with functional based proton planning using a structural‐based approach. We report the dosimetric comparison and estimate the reduction in radiation pneumonitis toxicity using a prior published Normal Tissue Complication Probability (NTCP) model developed directly from clinical plans and the dose values to the high functioning lungs.

## 2 METHODS AND MATERIALS

### 2.1 Patient characteristics and selection

This study was approved by the institutional review boards at University of Colorado, Denver (IRB #14‐1586) and Beaumont Health System (IRB #2016‐037). All patients included in the study provided written informed consent. The cohort included 31 patients who have stage I–IV or recurrent SCLC and NSCLC treated with definitive radiation therapy (45–60 Gy). Table 1 presents the patient characteristics. The primary patient selection criterion was the presence of a significant ventilation defect based on the lung functional profile obtained through 4DCT imaging. Quantitatively, we divided each lung into superior, middle, and inferior regions and calculated the percent ventilation in each third of the lung. If a patient’s ventilation image showed a reduction of 15% or more in ventilation in any one of the lung regions, the patient was deemed eligible for functional based planning. All 31 patients included in this study had significant ventilation defects. Patients with homogenous lung function were excluded from the study. The maximal tumor amplitude was evaluated in MIM (MIM Software Inc., Cleveland, OH) using deformable image registration of the GTV contours from the 4DCT data. For irregularly shaped or large tumor volume, only the portion of the GTV with the largest motion amplitude was reported.

**Table 1:** Patient characteristics

Characteristics	Value or no. of patients
Median age at diagnosis	
Years (range)	64 (44–81)
Diagnosis	
SCLC	5
NSCLC	26
Stage	
IA	1
IIA	3
IIIA	19
IIIB	4
IV	4
Gender	
Female	20
Male	11
Mean prescribed dose, Gy [RBE] (range)	
SCLC	48 (45–60)
NSCLC	58.2 (50–60)
Tumor location	
LUL	8
LLL	5
RUL	12
RML	2
RLL	4
Median max. tumor motion, cm (range)	0.6 (0.2–2.1)

Abbreviations: LLL, left lower lobe; LUL, left upper lobe; NSCLC, nonsmall cell lung cancer; RBE, relative biological effectiveness; RLL, right lower lobe; RML, right middle lobe; RUL, right upper lobe; SCLC, small cell lung cancer.

### 2.2 4DCT‐based ventilation calculation

**Fig. 1:**
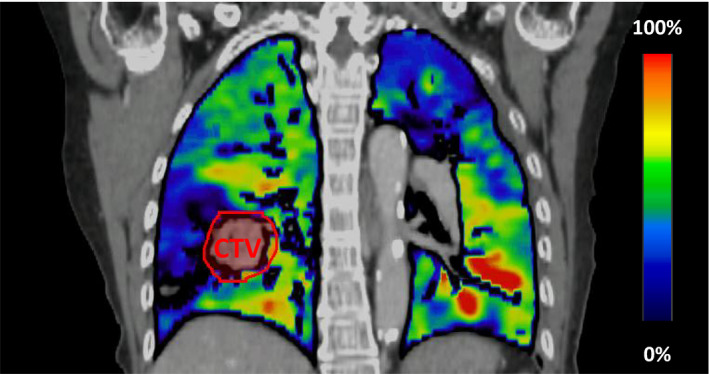
An example of a 4DCT‐derived pulmonary functional ventilation image overlaid with the averaged lung 4DCT data

The phase‐resolved pre‐treatment 4DCT simulation data from each patient was used to generate the pulmonary ventilation. Lung parenchyma was first segmented from the trachea, main bronchi, pulmonary vasculature, and the gross tumor volume on both the peak inhale and peak exhale phase data using intensity‐based segmentation algorithm. This segmented lung tissue mask determined the spatial domain in the inhale and exhale CT data where pulmonary ventilation was quantified. Based on the method proposed by Simon,^10^ the fractional changes in air content due to pulmonary ventilation in a specific CT voxel was represented by

(1) $$\frac{V_{\mathrm{in}}‐V_{\mathrm{ex}}\;}{V_{\mathrm{ex}}}=1000\frac{{\mathrm{HU}}_{\mathrm{in}}‐\;{\mathrm{HU}}_{\mathrm{ex}}}{{\mathrm{HU}}_{\mathrm{ex}}\left(1000+\;{\mathrm{HU}}_{\mathrm{in}}\right)},$$

where $V_{\mathrm{in}}$ and $V_{\mathrm{ex}}$ are the volumes of air within the inhale and exhale CT voxel pair respectively, and ${\mathrm{HU}}_{\mathrm{in}}$ and ${\mathrm{HU}}_{\mathrm{ex}}$ are the Hounsfield units of the mapped voxel pairs corresponding to the inhale and exhale breathing phases.^30^ Prior to the ventilation calculation, an intensity‐based quadratic penalty deformable image registration algorithm, with a spatial accuracy of 1.2 mm,^31^ was applied to establish spatial correspondence between the inhale and the exhale HU values pairs. The CT Hounsfield unit based ventilation calculation method, represented by eq. (1) was applied to each exhale CT voxel within the lung mask. The result was a 3D distribution of the normalized fractional change in voxel air content co‐registered to the peak exhale phase data. Figure 1 shows an example of the pulmonary ventilation generated using this workflow. According to eq. (1), for a given voxel in the exhale state, a fractional ventilation value of 0 means no change in volume between inhale and exhale. Such a voxel might be fixed in position with no displacement or they might only possess translational motion with no deformation between breathing states. A value of 1.0 means the initial volume has doubled going from exhale to inhale. The color scale map in Fig. 1 has the range of the RGB color spectrum from dark blue (value = 0) to red (value = max). The color scale is normalized to the maximum volume change and represented in percentage.

### 2.3 4DCT‐based functional avoidance treatment planning with volumetric modulated arc therapy (VMAT)

Although there are different methods for functional based treatment planning reported in the literature,^14^, ^32^, ^33^ this study employed the structural based treatment planning technique. In practice, a functional lung structure was auto‐segmented from the ventilation images by using a lower intensity threshold of 15%, which was normalized to the patient global maximum pixel intensity value.^34^ This functioning lung structure was further modified into different optimization structures to constrain the planning objectives for functional planning, while the standard planning does not consider functional structures. The photon data consisted of a standard lung plan and a clinically treated functional avoidance plan. Because the clinical trial involves two institutions, some of the patients were planned using the Varian Eclipse (Varian, Palo Alto, CA) treatment planning software with a True Beam machine and the rest of the VMAT plans were generated with the Pinnacle (Philips Healthcare) treatment planning system using an Elekta Agility (Elekta, Stockholm, Sweden). The gross tumor volume (GTV) was identified with contrast enhanced CT and/or PET scans. The internal gross tumor volume (iGTV) was designed to include the GTV motion in all phases of the 4DCT. The clinical target volume (CTV) was generated from an isotropic expansion of the iGTV by 6–10 mm. The planning target volume (PTV) was created by a 5 mm uniform expansion of the CTV. The planning constraints for the PTV and normal tissue structures generally followed the Radiation Therapy Oncology Group (RTOG) 0617 for NSCLC and RTOG 0538 for SCLC. To standardize the functional planning process between institutions, the planners were instructed to prioritize (1) Planning Target Volume (PTV) coverage, (2) meeting Organ at Risk (OAR) constraints, and (3) reducing dose to the functional lung structures.

### 2.4 4DCT‐based functional avoidance treatment planning with IMPT

The standard IMPT plans and functional avoidance IMPT plans were generated using the multi‐field optimization (MFO) method in the Eclipse Treatment Planning system v15.1 (Varian, Palo Alto, CA) with a 2.5 × 2.5 × 2.5 mm^3^ dose grid size and with two to three treatment fields per plan. A Hitachi Probeat‐V proton therapy system with 97 discrete proton energy levels were modeled and used for planning. This clinical machine model consists of a spot library with sigma in air (at isocenter) ranging from 5.7 mm at the lowest energy 71.3 MeV, to 2.5 mm at the highest energy 228.8 MeV, at the isocenter. In order to mitigate the dose degradation due to the interplay effect, a max MU iso‐layer spot repainting version of the machine model was used for planning.^35^ Instead of additional margin expansion from the CTV, range and setup errors were explicitly considered as part of the optimization problem to ensure robust CTV coverage. Robust proton plans were created with the Eclipse NUPO robust optimization algorithm. A setup uncertainty of 5 mm and a range uncertainty of 5% were chosen as the robust optimization parameters to account for patient daily setup errors and proton range uncertainties. During the plan robustness evaluation, for both the standard and functional avoidance plans, 95% of the CTV was required to be covered by 95% of the prescription dose in the worst‐case robustness scenario. If any of the worst‐case scenario plans doesn’t fulfill this clinical goal, the IMPT plan was re‐optimized with higher weighting on the CTV target coverage objectives to increase the plan robustness. As part of the routine plan check and quality assurance process, the proton plans have been re‐calculated using an in‐house GPU accelerated Monte Carlo 2nd check dose engine.^36^

### 2.5 Proton 4D interplay effect simulation

In order to estimate the 4D dynamic dose, an in‐house 4D simulation program was used to incorporate experimentally validated synchrotron proton spot scanning timing parameters^37^ and a GPU accelerated Monte Carlo dose calculation engine.^38^ The in‐house MC dose engine was benchmarked against GEANT4 simulations. The standard deviation of the disagreement of the models was found to be around 1% after accounting for the statistical uncertainties of both Monte Carlo techniques. The evaluation was carried out on a subset of the functional IMPT plans, selected based on tumor size and motion amplitude. Seven patients were chosen from the study cohort. The median and maximum tumor motion were 0.6 cm and 2.1 cm. Based on tumor motion, the patients chosen for simulation has a representative range of the following motion amplitudes: 0.4 cm, 0.7 cm, 0.8 c, 0.9 cm, 1.4 cm, 1.7 cm, and 2.1 cm. Also, patients were chosen based on their tumor volumes, the smallest tumor size was 108 cc and the largest tumor size was 834 cc. Proton dose was simulated and re‐calculated using this 4D dynamic dose pipeline to evaluate dose degradation caused by the interplay effect. The differences between the nominal Monte Carlo dose and the 4D dynamic dose for the following metrics were determined: D_5%_‐D_95%_ and D_95%_ of the CTV volumes, mean lung dose, mean esophagus dose, max cord dose, V20% and V30% of the functional lung, and the mean functional lung dose. A total of 6 simulations were performed for each patient by selecting 2 different breathing start phases (max inhale and max exhale) in combination with 3 different breathing periods (3, 5, and 7 s).

### 2.6 Dosimetric comparison and statistical analysis

A number of the CTV volume and organ at risk (OAR) dose metrics were evaluated for the comparison of the standard and functional VMAT and IMPT plans (see Table 2). For comparison reasons, the standard and functional IMPT plans, and the standard and functional VMAT plans, were all normalized to D99 of the CTV receiving 99% of the prescription. Comparison of the means of the dosimetric parameters was performed with the one‐way analysis of variance (ANOVA) with 0.05 selected as the alpha level. Post‐hoc paired student t‐tests with multiple testing correction were used to determine which groups demonstrated a difference in means.

**Table 2:** The comparison of DVH dose metrics between standard and functional treatment plans (*n* = 31) are presented for both VMAT and IMPT

	Volumetric Arc Therapy			Intensity Modulated Proton		
	Standard plan—Mean (SD)	Functional Plan—Mean (SD)	*P* value	Standard plan—Mean (SD)	Functional Plan—Mean (SD)	*P* value
CTV metrics						
CTV max (RBE[Gy])	70.93 (16)	71.45 (15.9)	0.1	62.09 (5.9)	62.86 (5.9)	0.001^a^
Conformity index	2.22 (0.7)	2.15 (0.7)	0.002^a^	1.79 (0.4)	1.71 (0.4)	0.001^a^
Homogeneity index	1.22 (0.3)	1.23 (0.3)	0.02	1.10 (0.2)	1.11 (0.2)	0.001^a^
OAR Metrics						
Total MLD (RBE[Gy])	16.14 (2.7)	15.28 (2.9)	<0.001^a^	9.73 (3.3)	8.78 (2.9)	<0.001^a^
Total lung V20 (%)	27.57 (5.7)	25.50 (6.1)	<0.001^a^	19.54 (6.8)	17.06 (5.7)	<0.001^a^
Total lung V5 (%)	66.8 (11.2)	64.89 (12.9)	0.005	29.80 (9.2)	27.67 (8.5)	<0.001^a^
Esophagus mean (RBE[Gy])	24.79 (8.2)	24.10 (8.6)	0.005	19.57 (10.0)	19.56 (10.1)	0.92
Heart mean (RBE[Gy])	11.90 (6.2)	11.88 (6.4)	0.92	4.62 (2.9)	4.77 (3.5)	0.75
Cord max (RBE[Gy])	36.18 (8.4)	36.72 (8.4)	0.46	31.84 (9.9)	33.57 (8.8)	0.1
Functional lung metrics						
Ipsilateral						
MLD (RBE[Gy])	24.38 (7.2)	22.49 (7.0)	<0.001^a^	17.65 (7.6)	14.72 (6.7)	<0.001^a^
Lung fV5 (%)	77.9 (17.6)	76.5 (18.3)	0.005	53.81 (18.2)	48.10 (17.9)	<0.001^a^
Lung fV20 (%)	51.6 (17.1)	45.57 (16.9)	<0.001^a^	35.83 (16.2)	28.05 (13.7)	<0.001^a^
Lung fV30 (%)	34.9 (16.1)	30.75 (14.8)	<0.001^a^	26.70 (14.2)	21.25 (12)	<0.001^a^
Contralateral						
MLD (RBE[Gy])	9.26 (3)	7.99 (2.7)	<0.001^a^	1.75 (2.4)	1.66 (2.2)	0.74
Lung fV5 (%)	69.6 (14.2)	66.08 (15.3)	0.015	8.24 (11.1)	6.88 (8.9)	0.002^a^
Lung fV20 (%)	5.79 (6.6)	4.10 (5.5)	<0.001^a^	2.68 (4.7)	1.66 (3.1)	0.004
Lung fV30 (%)	1.68 (3)	1.34(2.6)	0.013	1.02 (2.3)	0.75 (1.7)	0.015
Total lung						
MLD (RBE[Gy])	16.05 (3.3)	14.49 (3.3)	<0.001^a^	8.82 (3.9)	7.31 (3.3)	<0.001^a^
Lung fV5 (%)	73.7 (11.5)	71.06 (13.3)	0.003	28.78 (11.6)	25.40 (10.3)	<0.001^a^
Lung fV10 (%)	52.8 (13.7)	43.92 (12.8)	<0.001^a^	24.47 (10.4)	20.20 (8.4)	<0.001^a^
Lung fV20 (%)	26.38 (8.3)	22.66 (8.2)	<0.001^a^	17.59 (8.4)	13.48 (6.6)	<0.001^a^
Lung fV30 (%)	16.31 (6.9)	14.30 (6.6)	<0.001^a^	12.49 (6.9)	9.89 (5.7)	<0.001^a^
Lung fV40 (%)	10.54 (6.0)	9.28 (5.7)	<0.001^a^	9.01 (5.7)	7.32 (4.9)	<0.001^a^
Lung fV50 (%)	5.95 (4.2)	5.44 (4.2)	0.006	5.78 (4.1)	4.82 (3.6)	<0.001

Note

Functional lung metrics: Using the auto‐segmented functional lung structures from the calculated ventilation map. CTV = iGTV + Margin (Margin = 6 to 10 mm).

Abbreviations: fVX (%), functional %volume that receives X Gy; MLD, mean lung dose; OAR, organ at risk; RBE, relative biological effectiveness.

^a^ Statistically significant *P* value with multiple testing corrected alpha level 0.002.

### 2.7 Pulmonary toxicity analysis with functional lung NTCP model

Faught et al.^39^, ^40^ reported a functional lung NTCP model that shows strong predictive capacity for Grade 2+ and Grade 3+ radiation pneumonitis (RP), using the fractional volume of functional lung receiving 20 Gy, 30 Gy, and the mean functional lung dose (fV20 Gy, fV30 Gy, and fMLD). In an effort to quantify the possible toxicity reduction from 4DCT‐based functional avoidance plans, we applied their NTCP model and the dose metrics of the functional lung volumes to estimate the toxicity probability of the mean and its associated absolute reduction in Grade 2+ and Grade 3+ RP toxicity for both the photon and proton plans.

## 3 RESULTS

### 3.1 Dose statistics comparison between IMPT and VMAT

**Fig. 2:**
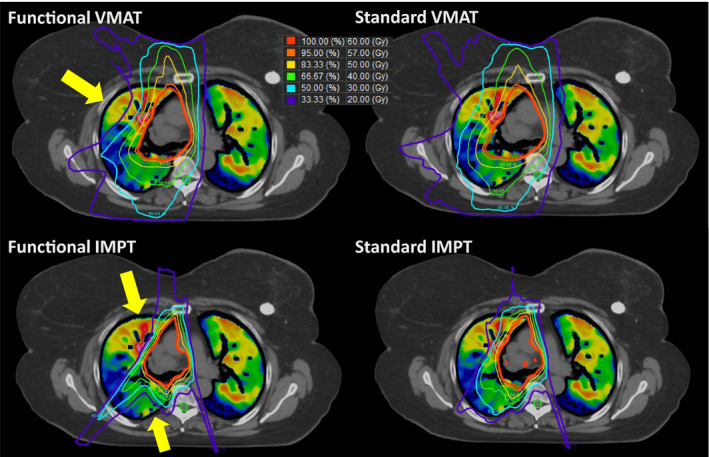
Examples of the standard and functional plans with VMAT (Top) and three‐field IMPT (Bottom) techniques. The yellow arrow indicates where the most functional sparing occurs for this particular patient

Figure 2 illustrates the isodose distribution of the standard and functional plans with VMAT and IMPT for one representative patient. The yellow arrows indicate where the most functional sparing occurred, given the sets of beam angles and optimization objectives used for this patient. Table 2 shows the mean and standard deviation of the dosimetric parameters as well as the *P* values for the corresponding *t* test. There are no significant dose differences observed for the esophagus, heart, and spinal cord between the standard and functional plans when comparing the same treatment modality. The functional IMPT plans show significant dose reduction when compared to the functional VMAT plans for the MLD at 15.28 Gy vs. 8.78 Gy, the lung V20 at 25.5% vs. 17.06%, mean esophagus dose at 24.1 Gy vs. 19.56 Gy, mean heart dose at 11.88 Gy vs. 4.77 Gy, cord max dose at 36.72 Gy vs. 33.57 Gy, fMLD at 14.49 Gy vs. 7.31 Gy, fV20 at 22.66% vs. 13.48%, and fV30 at 14.3% vs. 9.89%. When comparing the standard IMPT and the functional IMPT plans, the functional IMPT plans showed significant functional sparing to the fMLD from 8.82 (3.9) Gy to 7.31 (3.3) Gy, the fV10 from 28.78% (11.6%) to 25.4% (10.3%), and the fV20 from 17.59% (8.4%) to 13.48% (6.6%).

### 3.2 Interplay effect analysis on functional IMPT plans

**Fig. 3:**
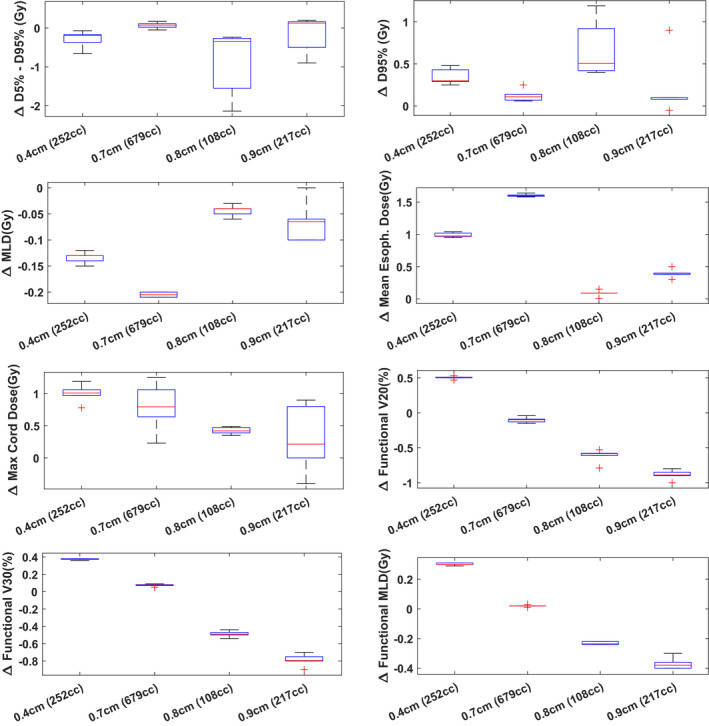
Dose metrics differences (nominal dose—4D dynamic dose) for a subset of lung patients (stages II to IV). A total of 6 single‐fraction interplay simulations were performed (combination of phase T0 start, phase T50 start, T = 3 s, T = 5 s, T = 7 s) per patient. Boxplots of the dose metrics differences are plotted here for each patient along with their maximum motion amplitude and tumor size

Figure 3 shows the DVH metric differences between the nominal Monte Carlo plans and the 4D dynamic plans. No significant dose difference was observed for the D_5%_‐D_95%_ and D_95%_ of the CTV volumes, mean lung dose, mean esophagus dose, max cord dose, V20% and V30% of the functional lung, as well as the mean functional lung dose. As a general observation, as the motion amplitude increases, there is increasing differences in the functional V20% and V30% of the lung, as well as the functional mean lung dose. The maximum observed difference in CTV D_5%_‐D_95%_ metric between the nominal plan and the 4D dynamic plan was 2.2 Gy. No significant dose degradation was observed for the CTV D95%.

### 3.3 Pulmonary toxicity reduction

Table 3 shows the estimated mean NTCP for Grade 2+ RP on three of the functional dose metrics and the calculated absolute toxicity reduction between functional and standard plans. There was statistically significant difference between the standard and the functional plans. An absolute reduction of 1.8%, 3.3%, and 5.7% was observed for fMLD, fV30, and fV20 dose metrics. For a certain patient, as high as 26% in absolute reduction was observed in Grade 2+ RP when planning with functional IMPT. We also report the Grade 3+ RP NTCP calculation results in Table A1 as an additional reference.

**Table 3:** Normal Tissue Complication Probability (NTCP) values for grade 2+ radiation pneumonitis are shown here for the average functional lung volume metrics for the standard and functional plans with both the VMAT and IMPT methods

Grade 2+ Toxicity			
Dose metric for NTCP	Toxicity probability of the mean (range)—VMAT standard	Toxicity probability of the mean (range)—VMAT functional	Absolute reduction (range)
fV20 Gy	31.3% (5.9%–67.8%)	25.1% (5.5%–60.2%)	6.2% (−0.1%–13.1%)
fV30 Gy	24.5% (9%–50.1%)	21.5% (8.5%–45.8%)	3% (0.1%–7.4%)
fVMLD	24.1% (11.9%–33.4%)	21.8% (11.4%–31.5%)	2.3% (−0.1%–5.5%)
Dose metric for NTCP	Toxicity probability of the mean (range)—IMPT Standard	Toxicity probability of the mean (range)—IMPT Functional	Absolute Reduction (Range)
fV20 Gy	17.6% (4.4%–51.3%)	12.7% (3.9%–36.1%)	5.70% (0%–26%)
fV30 Gy	19% (7.9%–47.5%)	15.7% (7.3%–35.9%)	3.30% (0%–15.3%)
fVMLD	14.6% (7.7%–25.4%)	12.9% (7.4%–21.3%)	1.80% (0%–5.9%)

## 4 DISCUSSION

The results presented in this study support the feasibility and the potential dosimetric gains of 4DCT‐derived functional avoidance IMPT planning for lung patients. By utilizing functional lung structures as additional optimization objectives, dose to the functional lung structures can be reduced without significant dose increases to the OAR structure. In contrast to the proton planning study performed by Huang et al.,^41^ we selected patient data from a prospective lung functional avoidance clinical trial with significant functional defects. We did not observe significant OAR dose increase when generating functional IMPT plans. We have also investigated the potential dose degradation caused by the scanning proton beam interplay effects.

It should be pointed out that one of the participating institutions (with 23 patients enrolled) permits dose escalation to the GTV with a 115% hotspot relative to the PTV dose. For some patients, the global maximum point dose could be as high as 130% of the PTV prescription. The IMPT plans were further generated by two different methods. The first method allowed the hot spot to exceed 130% of the prescription, while the second method required the strict adherence to our clinical proton lung treatment planning standard of practice (SOP), where the hot spot could not exceed 110%. Because of the differences in planning practice between institutions and modalities, we first performed dose metrics comparison of the OAR and functional lung structures between the dose‐escalated and non‐dose‐escalated proton plans. We did not see any significant DVH metric differences between the two methods except that some of the functional lung volume metrics show even lower values in the dose‐escalated planning methods. Knowing that the dose escalation will not introduce positive bias towards our comparison, we feel that it is justified to compare the dose‐escalated VMAT patients with their non‐dose‐escalated IMPT counterplans. We feel that it is important to compare treatment plans that are clinically relevant and adhere to the individual institution's planning standard of practice. The results of the comparisons are presented in Table B1.

Recently, a consensus statement has been published by the Particle Therapy Co‐operative Group (PTCOG) Thoracic Subcommittee,^42^ recommending that active motion management techniques, breath holding, beam gating, repainting, tracking, or adaptive planning should be used when significant motion (>5–10 mm) or changes in motion or anatomy occurs during treatments. Clinically, a number of these techniques are currently being utilized in our department to decrease the motion interplay effect in proton patients.^43^ The severity of the proton dose degradation caused by the interplay effect depends largely on proton therapy system parameters, such as the spot size, scanning magnet speed, dose rate, as well as the breathing amplitude, tumor size, and breathing regularity of a patient during treatment. Our 4D dynamic dose simulation showed that functional based IMPT plans were still robust against motion interplay for selected patients. Practical motion management strategies can be applied clinically for functional guided IMPT treatments. One shortcoming of our simulation on the interplay effect is that only one 4D dynamic dose simulation was performed for each patient data. Since it is generally believed that the dose interplay effect will be smeared out or lessened when we increase the number of treatment fractions. We believe our simulations will provide the worst‐case scenario where the entire prescription dose was delivered as a single fraction. However, the accuracy of the simulation still requires experimental validation with dosimetric measurements.

Additional analysis was also performed to investigate whether the amount of functional volume reduction was correlated to tumor volume or tumor location; unfortunately, there was no indication that the functional lung metrics correlates with tumor volume and location. Another important point is that the functional IMPT plans require the functional structures to be robustly spared. In other words, during the optimization of the IMPT plans, the functional lung structures must have no significant dose increase in the worst‐case scenario plans; the plan robustness curves or the robustness DVH band should be tightly grouped for the CTV volume, as well as for the functional lung structures. We would also like to point out that the NTCP functional model was generated with photon clinical lung plans and may not be entirely accurate for proton lung plans in estimation of the toxicity probability. For future studies, we aim to explore and incorporate longitudinal functional changes into adaptive planning workflow using weekly verification 4DCT information, since the pulmonary function could change during radiation therapy for lung patients.

## 5 CONCLUSION

The current work presents a comprehensive planning study across multiple institutions to investigate IMPT planning as a means of further reducing pulmonary toxicity. On average, functional IMPT planning can further reduce the total fMLD by 7.2 Gy, the total fV20 by 9.2%, and total fV30 by 4.4% over functional VMAT planning without increasing OAR dose. Minimal dosimetric degradation was observed when motion interplay effect was simulated for selected patients. NTCP calculation also shows that the risk of pulmonary complications can be further reduced with functional based IMPT for some patients.

## CONFLICT OF INTEREST

No conflict of interest.

## Data Availability

The data that support the findings of this study are available from the corresponding author upon reasonable request.

## 1

**Table A1:** Normal Tissue Complication Probability values for grade 3+ radiation pneumonitis are shown here for the functional lung volume metrics for the standard and functional plans in both the proton and photon modalities

Grade 3+ toxicity			
Dose metric for NTCP	Toxicity probability of the mean—VMAT standard	Toxicity probability of the mean—VMAT functional	Absolute reduction [range of reduction]
fV20 Gy	15.5% (2.9%–40.4%)	12.1% (2.7%–34.2%)	3.4% (−0.1%–8.1%)
fV30 Gy	11.3% (3.6%–27.8%)	9.7% (3.4%–24.7%)	1.6% (0%–4.4%)
fVMLD	11.1% (4.9%–16.4%)	9.8% (4.7%–15.3%)	1.3% (0%–3%)
Dose metric for NTCP	Toxicity probability of the mean—IMPT standard	Toxicity probability of the mean—IMPT functional	Absolute reduction [range of reduction]
fV20 Gy	8.4% (2.2%–27.7%)	6% (2%–18.1%)	2.4% (0%–15.4%)
fV30 Gy	8.4% (3.1%–25.9%)	6.7% (2.9%–18%)	1.7% (0%–10.2%)
fVMLD	6.2% (3.1%–11.8%)	5.4% (2.9%–9.6%)	0.8% (0%–3.2%)

**Table B1:** DVH dose metrics comparisons between non‐dose‐escalated and dose‐escalated IMPT plans (*n* = 22)

	Intensity modulated proton—standard			Intensity modulated proton—functional		
	Nondose‐escalated plan	Dose‐escalated Plan	*P* value	Nondose‐escalated Plan	Dose‐escalated Plan	*P* value
CTV metrics						
CTV max (RBE[Gy])	63.82	74.36	<0.00	64.64	74.36	<0.00
OAR Metrics						
Total MLD (RBE[Gy])	10.71	10.97	0.37	9.72	9.97	0.43
Total lung V20 (%)	21.26	20.99	0.84	18.60	18.02	0.39
Total lung V5 (%)	30.77	30.24	0.48	28.77	28.76	0.99
Esophagus mean (RBE[Gy])	19.04	20.34	0.33	18.97	20.51	0.23
Heart mean (RBE[Gy])	5.38	4.78	0.12	5.06	4.86	0.38
Cord max (RBE[Gy])	32.56	35.06	0.23	34.38	35.93	0.37
Functional lung metrics						
Ipsilateral						
MLD (RBE[Gy])	21.210	21.213	0.7	17.90	17.81	0.88
Lung V5 (%)	60.86	58.44	0.004	55.19	53.74	0.17
Lung V20 (%)	42.78	41.45	0.51	34.08	31.52	0.053
Lung V30 (%)	33.17	32.86	0.94	26.79	24.71	0.08
Contralateral						
MLD (RBE[Gy])	1.91	1.85	0.85	1.90	1.21	0.11
Lung V5 (%)	8.47	7.93	0.39	7.04	6.80	0.7
Lung V20 (%)	3.02	2.69	0.4	1.82	1.26	0.11
Lung V30 (%)	1.26	1.05	0.4	0.92	0.64	0.17
Total lung						
MLD (RBE[Gy])	10.17	10.18	0.68	8.52	8.38	0.52
Lung V5 (%)	31.10	29.96	0.03	27.82	27.13	0.22
Lung V10 (%)	27.11	25.89	0.02	22.55	21.81	0.18
Lung V20 (%)	20.26	19.65	0.52	15.80	14.43	0.017
Lung V30 (%)	15.09	14.80	0.84	12.15	11.12	0.02
Lung V40 (%)	11.33	11.02	0.58	9.43	8.83	0.08
Lung V50 (%)	7.61	7.73	0.52	6.46	6.48	0.95
